# Revealing genetic links of Type 2 diabetes that lead to the development of Alzheimer’s disease

**DOI:** 10.1016/j.heliyon.2022.e12202

**Published:** 2022-12-16

**Authors:** Muhammad Afzal, Khalid Saad Alharbi, Sami I. Alzarea, Najiah M. Alyamani, Imran Kazmi, Emine Güven

**Affiliations:** a Department of Pharmacology, College of Pharmacy, Jouf University, Sakaka 72341, Aljouf, Saudi Arabia; b Biology Department, College of Science, University of Jeddah, Jeddah, Saudi Arabia; c Department of Biochemistry, Faculty of Science, King Abdulaziz University, Jeddah, Saudi Arabia; d Department of Biomedical Engineering, Düzce University, Düzce, Turkey

**Keywords:** Alzheimer’s disease, Type 2 diabetes mellitus, Neovascular unit, Differential expression, Ageing brain

## Abstract

**Background:**

A factor leading to Alzheimer’s Disease (AD), portrayed by peripheral insulin resistance, is Type 2 diabetes mellitus (T2D). The likelihood of T2D cases would be at boosted danger in alternating AD cases has severe social consequences. Several genes have been detected via gene expression profiling or different techniques; despite the consideration of the utility of numerous of these genes stays insufficient.

**Methods:**

This project is designed to uncover the mutual genomics motifs between AD and T2D via non-negative matrix factorization (NMF) of differentially expressed genes (DEGs) of T2D Mellitus of human cortical neurons of the neurovascular unit gene expression data. A rank factorization value is calculated by employing the combination of the NMF model with the unit invariant knee (UIK) point method. The metagenes are further determined by remarking the enriched Kyoto Encyclopedia of Genes and Genomes (KEGG) pathway and gene ontology (GO) enrichment tools. In this study, the most highly expressed genes of metagenes are subjected to protein-protein interaction (PPI) network study to discover the most significant biomarkers of T2D Mellitus in the ageing brain.

**Results:**

We screened the most important shared genes (CDKN1A, COL22A1, EIF4A, GFAP, SLC1A1, and VIM) and essential human molecular pathways that motivate these diseases. The study aimed to validate the most significant hub genes using network-based methods which detected the corresponding relationship between AD and T2D.

**Conclusions:**

Using in silico tools, the computational pipeline has broadly examined transformed pathways and discovered promising biomarkers and drug targets. We validated the most significant hub genes using network-based methods which detected the corresponding relationship between AD and T2D. These consequences on brain cells hypothetically reserve to diabetic Alzheimer’s so-called type 3 diabetes (T3D) and may offer promising methodologies for curative intrusion.

## Highlights

• The study extracted the most significant genes in common of T2D and AD such as CDKN1A, COL22A1, EIF4A, GFAP, SLC1A1, and VIM.
• The hub genes of AD-related genes were validated using network-based methods.
• Notional genetics mechanisms of T2D involvement in AD etiology are presented.

## 1 Introduction

As the primary Rotterdam project reports a growing threat to the development of Alzheimer’s disease (AD) in individuals with Type 2 diabetes mellitus (T2D), a quantity of medical and clinical reports have specified additional straight proof to reinforce the association between T2D and AD. These diseases are both related to aging, and each rises the possibility of the evolvement of the other. Medical, epidemiologic, imaging, and biochemical studies have demonstrated that raised glucose levels and diabetes are linked with cognitive dysfunction, the most documented reason for which is Alzheimer’s disease [1, 2].

T2D may directly damage brain neurovascular unit cells besides its contribution to vascular diseases such as peripheral artery, abdominal aortic aneurysm, and carotid artery diseases. The likelihood of T2D cases would be at boosted danger in alternating AD cases has severe social consequences. More than 30 million people live worldwide and 5.3 million individuals in the US with AD. As the population ages, those numbers are projected to grow significantly over the next decade. The direct and indirect expenses related to AD in the United States (US) solely has surpassed 600 billion US dollars yearly [3]. The threat of AD for T2D is far grimmer, likewise, of over 23 million T2D cases in the US, ≈18 million may be up to 50% more at threat of developing AD than non-diabetics [4].

A cohort study of 10,095 partakers in the United Kingdom (UK) recorded a sum of 639 cases of dementia and 1710 cases of diabetes. It was stated that diabetes occurring at an early age was significantly linked with the progression of dementia. Though, the motivating machinery liable for this linkage still remains unclear [5].

Rising indication from the latest conclusions shows that T2D and AD have common molecular machinery, for example, oxidative stress [6], hyperglycemia [7], and apoptosis [8]. Bioinformatics examination of genomics has been influential in uncovering common biological machinery of comorbid disorders. Numerous findings based on whole gene expression analysis also maintain the common pathogenesis of T2D and AD. Shu et al. (2022) noted that complement dysregulation performs key positions in the pathogenesis of T2D and AD. One recent study explored blood transcriptomic patterns between T2D and AD and uncovered genome components that were dysregulated in both AD and T2D [9]. Another study utilizing a non-negative matrix factorization method (NMF) classified the latent relationship of T2D and AD in the framework of gene expression datasets and identified pathways linked with shared clinical traits of T2D and AD involving chemokine signaling and immune system-associated pathways [10]. These results pointed out that T2D might take an essential role in developing dementia and in each stage of AD through distinct biological, functional, and molecular pathways [11].

Latest reports have proven that diseases cannot be developed by just a single gene, feature, or tissue. There must be a connection between different genes, proteins, biological, signaling, functional, and cellular pathways that act together like an orchestra [12, 13]. The NMF is a procedure that clusters genes with similar expression subsets in common gene expression and those with different gene expression in different units. Various research has demonstrated the non-negative matrix factorization can be utilized to explore genes of interest in diverse cells and tissues [10, 14, 15, 16].

The large dimensionality of big datasets developed by DNA microarray technology requires to be aggregated for clustering and visualization [17]. The clustering approach is beneficial in the perception of indefinite gene-gene relationships [18]. Genes or proteins with the same expression profiles are initially arranged and then clustering trees are built in the hierarchical clustering technique [19]. The restraints of hierarchical clustering are its large capacity to generate an invariant clustering tree and its responsive structure to similarity measures [20]. Thus, several bi-clustering approaches have been advanced to handle the disadvantages of the conventional clustering techniques stated beyond. The principal component analysis (PCA), independent component analysis (ICA), and non-negative matrix factorization (NMF) are common bi-clustering methods that can utilize instantaneous classes of genes structured beneath distinctive situations [21, 22]. The utilization of the PCA depends on the linearity presumption of the microarray gene expression datasets [23]. The NMF is a bi-clustering approach that factors into two distinct non-negative matrices and is a superior alternate of others. It was originally employed in image recognition study [24] and has been broadly used in bioinformatics research such as gene expression, sequencing analysis, proteomics, and metabolomics in latest years [25, 26, 27, 28]. The NMF is a dimension-reduction method that transforms big expression data from thousands of proteins (genes) to signatures or so-called metagenes. It has been used extensively for almost two decades since it is less sensitive to filter genes and screen numerous traits of expression data [15]. Furthermore, it is used in numerous bioinformatics topics such as cross-platform classification, class comparison, studying functional genes heterogeneity, and molecular pattern discovery [29]. From computation point, PCA principally depends on experimental covariance matrix in which many samples are necessary. However, in gene expression data, the number of samples is always smaller than a hundred besides much smaller than the number of genes (in thousand scale) which demonstrates the superiority of NMF.

A fresh study [30] reported alterations in cortical neurones involved changes in insulin, insulin-related signaling pathways, cellular senescence, inflammatory regulators, compartments of the mitochondrial respiratory electron transport chain, and cell cycle utilizing the co-expression technique. Bury et al., 2021 further demonstrated that reduced insulin signaling was common in neurovascular unit cells with, also, apoptotic pathway modifications in astrocytes and dysregulation of advanced glycation end-product signaling in endothelial cells.

This study aims to identify genes of T2D that leads to AD that are not identified by other hierarchical clustering techniques [31]. Earlier studies proposed that NMF is an effective instrument to obtain biological info from the microarray data and comprehending T2D expression data and its link with Alzheimer’s disease [10, 32, 33]. To increase the deep information expression dataset, the DEGs analysis method has been widely performed using student’s t-test and its versions [34]. Hence, in this current study, our ultimate aim is to develop biologically related understanding and advance more perceptions into the pathobiology of T2D and its link with AD by utilizing the NMF on DEGs of the GSE161355 dataset.

The study first identified differentially expressed genes (DEGs) using the limma package of R studio. To advance further insights about the progression of T2D and its association with AD, we operated the NMF algorithm to identify the metagenes convoyed by clinical features of the DEGs. Employing the combination of the NMF model with the UIK technique, this study identified metagenes, patterns of differentially expressed genes with correlated expression alterations in essential human molecular pathways linked with T2D that may be an underlying cause of neuronal damage and dysfunction. The gene ontology terms analysis and KEGG pathway investigation were implemented on the hub genes. We further used these genes to build the PPI network to identify the common hub genes. We then studied and identified DEGs of the GSE54765 [35] Alzheimer’s disease microarray gene expression data to validate the mutual gene targets on T2D hub genes.

We further inspected the authentication of our computational pipeline utilizing the gene-disease association (GDA) network and the common hub genes-transcription factors interaction (TFI) network. These network-based methods screened the most significant common pathways which motivate these diseases. Overall, this study detected the critical involvement of the most significant in the biological, cellular, and functional pathways that might explain neuronal damage and dysfunction, hypothetically causing diabetic AD.

## 2 Materials & methods

### 2.1 The gene expression datasets

The Type 2 Diabetes (T2D) Mellitus of human gene expression dataset was retrieved from NIH GEO database (https://www.ncbi.nlm.nih.gov/geo/query/acc.cgi?acc=GSE161355) [30]. The dataset includes the total RNAs from Neurone, astrocyte, and endothelial cell-enriched, performed on 6 cases with T2D in a neuropathology cohort, and 5 age and sex-matched. The microarray platform includes data from 5 control and 6 diabetics of astrocytes, endothelial cells, and neurons of each respectively in a total of 33 samples. The GSE54765 gene expression dataset [35] includes the experimental data on the genes regulated by cells cultured without (control) and with (treatment) 1,25-dihydroxyvitamin D3 brain pericyte cells which have been extracted from a neuron and glial cells. To screen the DEGs of the GSE54765, four samples of two replicates for control samples and two replicates treated with 1,25D3 were defined as groups.

The probe annotation files of both datasets were GPL570 platform which mapped all the Affymetrix probe ids to the gene symbols, and gene expression values were log2 transformed. As a result, samples of gene expression datasets obtained genomic information ranging. Both of the datasets were studied exploratory utilizing the GEOquery package of Bioconductor under conventional modes in R programming [36, 37, 38, 39]. Further software packages used are Biobase, biomaRT, umap and gplots [37, 38, 40, 41]. Toward determining the adjusted p-value and preventing Type I errors, this study utilized the method of Benjamini-Hochberg. A hypergeometric standard was done on the down-regulated and up-regulated DEGs to extract significant GO and KEGG pathway enrichments and calculated a false discovery rate (FDR) [42, 43].

### 2.2 DEGs as the target matrix of the NMF

**Figure 1:**
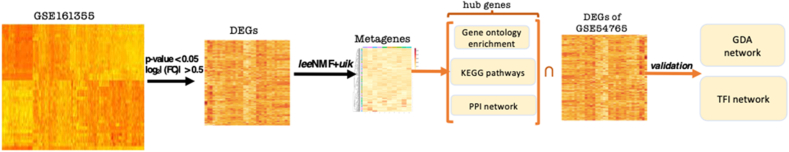
Design of the computational pipeline. The UIK based method for extraction of metagenes between control and T2D cells. The FC, fold change; DEGs, differentially expressed genes; GDA, gene-disease association; TFI, transcription-factor interaction.

Analysis and statistical computations of the GSE161355 dataset is conducted in R programming version 3.6.3 [44]. Figure 1 demonstrates the study design and workflow. The probes with the low number of reads were screened initially to improve DEGs detection sensitivity [45]. The rest of the expression values are converted to a logarithmic measure. The samples are separated into two sets providing control (neurones, astrocytes, and endothelial cells) and T2D cells [10]. Biomart package is utilized to annotate probes to official gene symbols of DEGs (Durinck et al., 2005, 2009). For repeated official gene symbols, the expression values of the identical official gene symbol were combined into an average number. We selected the DEGs in the R package “limma” [46] at p−value<0.05 and |log(FC)|>0.5 from GSE161355 dataset. Likewise, DEGs of the GSE54765 is selected at the significance p−value<0.05 and |log(FC)|>0.5 shown in Figure 1.

### 2.3 The non-negative matrix factorization model based on UIK method

The NMF algorithm was presented by Lee and Seung, 1999 [24]. It is classified in the same group in terms of dimension reduction algorithms of unsupervised learning models for instance factor analysis (FA), vector quantification (VQ), and principal component Analysis (PCA).

The NMF model allows a regional, piece-centered illustration of the reports contrary to the more comprehensive facts portrayal generated by PCA, FA, and VQ techniques. It reveals metagenes formed by correlated genes that drawing a local gene expression structure as performed on a microarray dataset. Unlike established clustering techniques such that k−means and hierarchical clustering approaches, genes can occur in several metagenes but are not limited by the formula as a component of a single cluster. The model further provides genes in diverse clusters at distinct revival levels rather than in only one big cluster. These characteristics allow the NMF algorithm to be a perfect approach for analyzing the GEO/GSE datasets. Previously, the NMF method was performed on the brain cancer and leukemia gene expression datasets to provide cancer subtypes and to find metagenes [15].

The aim is to factorize the target matrix V into a trivial number of rows, r so-called “rank” and all described as a positive linear combination of the V matrix itself. This study incorporated the NMF model to find best possible rank utilizing the Unit Invariant Knee (UIK) method [47, 48] on DEGs of ageing brain. The suggested UIK technique is free of a priori rank value to enter, straight to achieve, and does not demand primary constraints that significantly impact functionality of the model [48, 49, 50, 51]. To reduce the dimension of the microarray dataset significantly and to describe the discrepancies between features, NMF was applied using an R package called “NMF” [52]. Furthermore, investigation on genes with rationally sizable factors in every genetic and operative procedure may offer some advantages, supposedly that complementary genes may act in excess of one biological course. The particular metagene genes were screened employing Kim and Park’s scoring method [53] in our study [52, 54].

### 2.4 Enrichments of the gene ontology (GO) terms of metagenes

The measurement annotations of expression levels for probes of metagenes were corresponded official gene symbols through the Biomart package in the R language (R Core Team, 2020). The characterization of the differentially expressed genes of biological processes (BC), molecular functions (MF), and cellular components (CC) of the GO study was investigated by the **D**atabase for **A**nnotation, **V**isualization, and **I**ntegrated **D**iscovery (**DAVID**) [55]. After the metagenes extraction with the correlation coefficient scale, the characterization of different parts such as Universal Protein source, physical and biological properties such as GO, and annotation terms operating DAVID and KEGG (Kyoto Encyclopedia of Genes and Genomes) was studied [56, 57].

### 2.5 The human protein-protein interaction (PPI) network of metagenes

NetworkAnalyst [58] offers the construction of the PPI networks of a single gene list as an input through the STRING Interactome. To reveal the regulative machinery of metagenes classes of normal and Type 2 diabetes gene expression dataset was analyzed to build a PPI network with the noted GO terms.

### 2.6 Validation of the common hub genes

#### 2.6.1 Analysis of an Alzheimer’s disease gene expression dataset

This study retrieved another human Alzheimer’s Disease (AD) from the NIH Gene Expression Omnibus (GEO) by searching the words “Alzheimer’s”, “affy”, and “human” in the GEO public database. The GSE54765 microarry gene expression dataset [35] composes of the experimental data on the genes regulated by cells cultured without (control) and with (treatment) 1,25-dihydroxyvitamin D3 brain pericyte cells which have been extracted from the neuron and glial cells. Four samples of two replicates for control samples and two replicates treated with 1,25D3 were studied. The DEGs of the GSE54765 dataset was filtered standalone. Specifically, we focused on the expression levels of key biomarkers from the AD gene expression dataset to confirm the importance of biomarkers in the T2D dataset of the aging brain.

#### 2.6.2 The gene-disease association network of the common hub genes

This study built a gene-disease network to verify the connection of the shared genes (T2D and AD datasets) using NetworkAnalysist. The information was collected via the network link through the DisGeNET database [59], which is a widely used database relating information on human disease-associated genes and deviations.

### 2.7 The common hub genes – transcription factor (TF) interaction network

This project used the JASPAR database [60] via NetworkAnalyst to detect TFs based on the regulatory transcriptional level of common hub genes. The network topological parameters such as betweenness centrality and degree were calculated to rank the classified TFs. The database are employed on collected genes of entire acknowledged diseases, and genotype-phenotype association to validate the norm of the TFI network-based methodology.

## 3 Results

### 3.1 The gene expression data analysis of T2D

This study used the Type 2 Diabetes (T2D) Mellitus dataset consisting of 33 Affymetrix Human Genome U133 Plus 2.0 Array samples (six cases with patient-reported T2D, and five age and sex-matched controls in separate experimental series) conducted on the human brain neuron, astrocyte, and endothelial cells. The study demonstrated (UMAP) uniform manifold approximate and projection for quality control (QC) and a boxplot and hierarchical clustering of gene expression samples in Figure S1ABC respectively.

We selected 998 DEGs (Figure S1D) such that 780 were down-regulated and 218 were up-regulated genes in the R package “limma” [46] at p−value<0.05 and |log(FC)|>0.5 from the GSE161355 dataset. The DEGs list is available at Table S1.

### 3.2 The decision of the optimal rank parameter for the NMF model via UIK method

A significant task of the NMF is reducing the dimensionality of the target matrix of differentially expressed genes space to a considerably less dimension of *r*. So, this study decided the optimum rank parameter *r*, which factors the expression dataset into *r* metagenes or encoding measurements. In Figure S2A, the Residual Sum of Squares (RSS) is drawn against different *r* values in the interval [3, 30].

The rank value of the RSS curvature is in among knee points located via the uik () function at *r* = 10. This plot-building methodology was adapted from the research of Christopoulos et al., 2016, as designated in a former study [48]. By employing the combination of the NMF model with the UIK method, this study identified ten metagenes, patterns of differentially expressed genes with correlated expression.

### 3.3 The analysis of the metagenes

Any metagene depicts a bunch of genes acting in a practically correlated form in the genome. To achieve the dimension-reduction of the DEGs to a collection of metagenes and linked to encoding measurements, the analysis of the Type 2 Diabetes Mellitus gene expression dataset engaged employing the NMF method. Furthermore, the metagenes reveal the activation level of each encoding measurements on a particular cell feature (signature) inside the DEGs.

The metagenes analysis grouped the DEGs within each metagene in reducing order and the metagenes in descending order as reported by the activation levels of their genes. Metagene 2 and Metagene 9 represent the highly expressed metagene within the DEGs, whereas Metagenes 3, 5, and 10 illustrate the smallest favorably expressed. Specific DEGs of the metagenes have been neglected, which contained less than two DEGs, or have not been annotated at all. Table 1 demonstrates the DEGs included in Metagene 9. A list of all the metagenes with the top 15 DEGs can be found in Table S2.

**Table 1:** A significant genes for Metagene 9.

Probe ID	Transcript	Official Gene Symbol	Expression	logFC	Chromosome	Description
206547_s_at	X	PPEF1	4.62719897	0.857	9	protein phosphatase with EF-hand domain 1
201693_s_at	5	EGR1	7.73370161	0.633	1	early growth response 1
226273_at	X	CLCN5	6.10922621	0.694	9	chloride voltage-gated channel 5
213664_at	9	SLC1A1	5.444564	0.855	3	solute carrier family 1 member 1
1552742_at	3	KCNH8	3.6104217	0.578	3	potassium voltage-gated channel subfamily H member 8
200940_s_at	1	RERE	6.28630339	0.757	20	arginine-glutamic acid dipeptide repeats
228873_at	8	COL22A1	4.69490112	0.85	7	collagen type XXII alpha 1 chain
213921_at	3	SST	5.10968027	0.713	1	somatostatin
235007_at	4	BBS7	6.12447676	0.688	6	Bardet-Biedl syndrome 7
223678_s_at	10	SFTPA1	9.29410188	0.656	5	surfactant protein A1
214985_at	8	EXT1	7.60148361	0.694	5	exostosin glycosyltransferase 1
2 231787_at	6	SLC25A27	7.68224312	0.691	8	solute carrier family 25 member 27
B. The significant genes for Metagene 2.						
Probe ID	Transcript	Official Gene Symbol	Expression	logFC	Chromosome	Description
201160_s_at	12	YBX3	4.988485697	-0.844	18	Y-box binding protein 3
220016_at	11	AHNAK	8.069689818	-0.691	8	AHNAK nucleoprotein
201426_s_at	10	VIM	7.552385515	1.14	10	vimentin
203540_at	17	GFAP	5.456329758	0.935	38	glial fibrillary acidic protein

### 3.4 Metagene involvement in the temporal cortex of type 2 diabetes gene expression dataset

**Figure 2:**
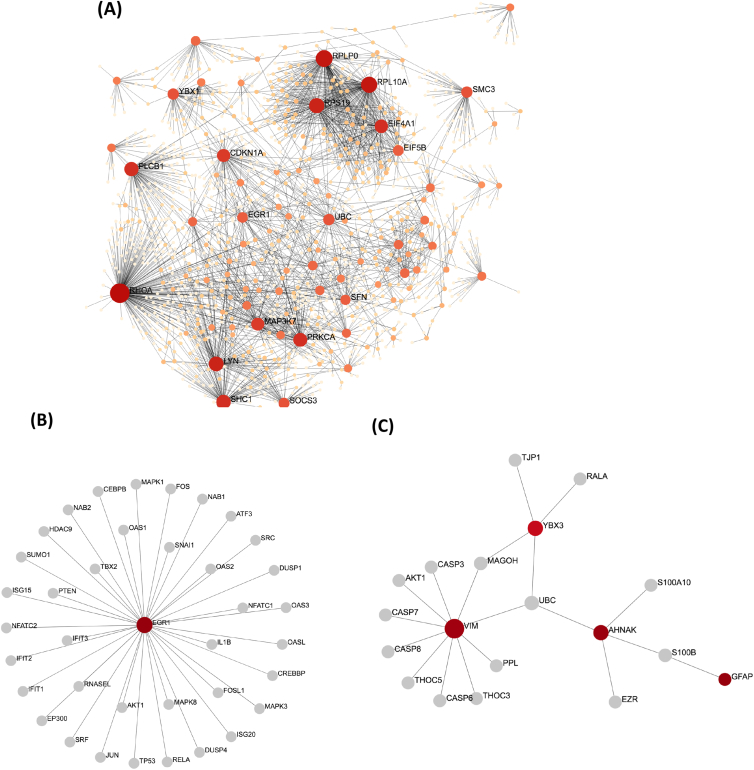
(A) The PPI network of DEGs of gene expression of Type 2 Diabetes data detected by NetworkAnalyst. (B) The network is illustrated in the Metagene 9-PPI network. (C) The network displayed is the Metagene 2-PPI network. The colors present the expression levels of the genes. Specifically, the red to light orange indicates the nodes (genes) highly expressed to lowly expressed. The domains of the nodes present the degrees to which the nodes bond with others. The grey-colored connections are not the DEGs themselves but have a tight bond with (A), (B), and (C) red nodes. Finally, the node sizes describe the level of significant genes degree centrality, i.e., the more prominent amount of neighbors a node has.

One excessive pinpoint in Figure 2B is that metagenes 2 and 9 are the arch metagenes affected in the highly expressed genes and is evident to silence the other metagenes. These metagenes further describe the most assiduously expressed metagenes our investigation revealed. The actively expressed DEGs of these metagenes are subject to examine functional annotations studied via DAVID (http://david.abcc.ncifcrf.gov/). Metagenes 2, 3, 5, and 10 are primarily involved in the Jak-STAT cascade, epidermal growth factor receptor signaling pathway, and response to endogenous stimulus biological processes respectively as shown in Table 2. The most significantly enriched gene ontology terms for Metagene 9 are presented in Table 3. Metagene 2 is involved in focal adhesion kinase activity (GO:0004715), SH2 domain binding, and protein tyrosine kinase activity which regulates cellular proliferation, survival, adhesion, and differentiation, and their role is thus strictly regulated. Metagene 9 is involved in ion transport and ion binding, ion metal-binding pathways whereas Metagene 5 is primarily involved in protein kinase binding and cytosol. Although all the other metagenes are stifled, another characteristic of Tables 2 and 3 together is that Metagenes 2, 3, 5, 9, and 10 come out very active in the KEGG pathways linked to the Jak-STAT signaling pathway, protein processing in endoplasmic reticulum cellular senescence, carbohydrate digestion and absorption, and insulin signaling pathway respectively.

**Table 2:** The Most Enriched GO and KEGG enrichment categories for Metagenes 2, 3, 5, and 10.

Category	Term	PValue	FDR	Metagene
KEGG	Jak-STAT signaling pathway	6.23E-27	1.98E-24	2
BP	Jak-STAT cascade	8.04E-24	6.59E-21	2
MF	Non-membrane spanning protein tyrosine kinase activity	3.64E-15	1.41E-12	2
CC	Cytosol	9.75E-13	2.19E-10	2
KEGG	Protein processing in endoplasmic reticulum	6.00E-04	6.36E-04	3
BP	Response to unfolded protein	1.08E-06	2.48E-04	3
MF	Unfolded protein binding	3.49E-07	2.20E-05	3
CC	Extracellular region part	2.76E-02	1.82E-02	3
KEGG	Cellular senescence	2.10E-17	6.68E-15	5
BP	Epidermal growth factor receptor signaling pathway	1.45E-20	1.19E-17	5
MF	Protein kinase binding	2.52E-15	9.79E-13	5
CC	Cytosol	1.58E-19	3.55E-17	5
KEGG	Insulin signaling pathway	1.51E-13	4.80E-12	10
BP	Response to endogenous stimulus	1.34E-14	7.32E-13	10
MF	Phosphatase binding	3.12E-07	8.63E-06	10
CC	Protein complex	0.000477	0.00677	10

**Table 3:** Metagene 9 involvement in KEGG Pathway enrichment and three gene ontologies (biological process, cellular component, and molecular function).

Category	Term	Count	%	PValue	Genes
KEGG_PATHWAY	hsa04974: protein digestion and absorption	2	18.1	0.00500832	COL22A1, SLC1A1
GOTERM_BP_FAT	GO:0007420∼brain development	3	27.2	0.00376687	EXT1, RERE, BBS7
GOTERM_BP_FAT	GO:0006811∼ion transport	4	36.3	0.01563589	SLC25A27, CLCN5, KCNH8, SLC1A1
GOTERM_BP_FAT	GO:0055085∼transmembrane transport	3	27.2	0.02524481	CLCN5, KCNH8, SLC1A1
GOTERM_BP_FAT	GO:0051049∼regulation of transport	3	27.2	0.00272881	SLC25A27, CLCN5, KCNH8
GOTERM_CC_FAT	GO:0045177∼apical part of cell	3	27.2	0.00330427	SLC25A27, CLCN5, SLC1A1
GOTERM_CC_FAT	GO:0031226∼intrinsic component of plasma membrane	3	27.2	0.00379435	CLCN5, KCNH8, SLC1A1
GOTERM_CC_FAT	GO:0005783∼endoplasmic reticulum	3	27.2	0.00414683	EXT1, COL22A1, SFTPA1
GOTERM_CC_FAT	GO:0044421∼extracellular region part	4	36.3	0.00423322	SST, COL22A1, SLC1A1, SFTPA1
GOTERM_MF_FAT	GO:0043167∼ion binding	5	45.4	0.00743422	EXT1, RERE, CLCN5, SLC1A1, PPEF1
GOTERM_MF_FAT	GO:0046872∼metal ion binding	3	27.2	0.00341425	EXT1, RERE, PPEF1

### 3.5 GO pathway and KEGG enrichment analysis of Metagene 9

**Figure 3:**
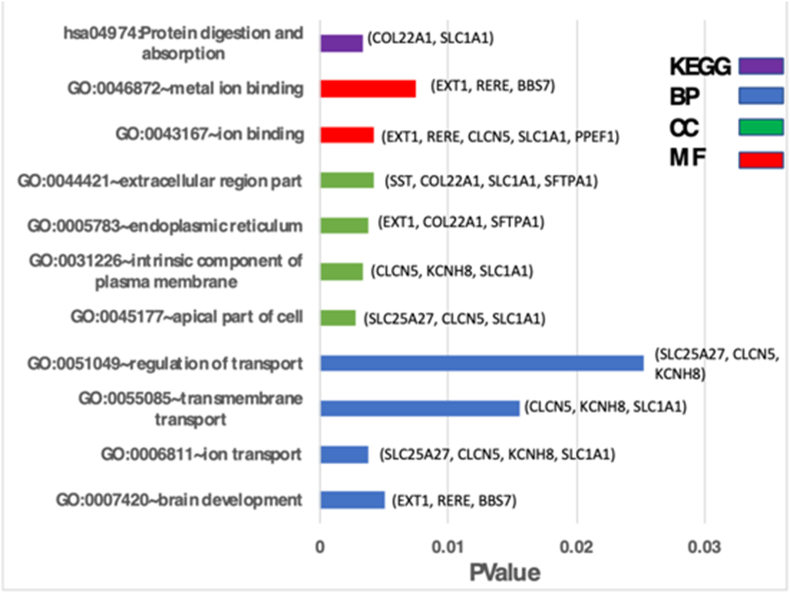
A bar plot of Metagene 9 involving KEGG pathway result and gene ontology (GO) terms of biological process (BP), cellular component (CC), molecular function (MF), and corresponding genes in each pathway. The x-axis indicates the pathways and the y-axis indicates the *p-value*.

Gene expression analysis using GO pathway enrichments on Type 2 Diabetes Mellitus Metagene 9 set reveals the first set of functional characterization in Figure 3 along with genes involved in each pathway. Table 3 demonstrates the significant enrichments of the Metagene 9 using biological processes (BP) brain development (GO:0007420), ion transport (GO:0006811), and transmembrane transport (GO:0055085). The most enriched genes in the cellular component (CC) contains the apical part of the cell (GO:0045177), intrinsic component of plasma membrane (GO:0031226), and endoplasmic reticulum (GO:0005783). Lastly, the significant enrichments of GO terms in molecular function (MF) are uncovered ion binding (GO:0043167) and metal ion binding (GO:0046872). KEGG pathway analysis illustrated that the DEGs involved in Metagene 9 were considerably enriched in hsa04974:protein digestion and absorption.

### 3.6 Metagenes 2, 3, 5, and 10

The study observed through Figure S2B that metagenes 2, 3, 5, and 10 were the primary metagenes tangled in the protein-related transport, binding, and regulation of gene ontology enrichments. A more intimate aspect in Table 2 for these metagenes is obvious to confirm this observation. As shown in Table 2, Metagene 2 is enriched in the Jak-STAT signaling pathway, Jak-STAT cascade, non-membrane spanning protein tyrosine kinase activity, and cytosol. Moreover, Metagene 3 is heavily in contact with protein processing, unfolded protein response, unfolded protein binding, and extracellular region fragment in the endoplasmic reticulum. Metagene 5 is involved in cellular senescence, epidermal growth factor receptor signaling pathway, protein kinase binding and cytosol. Metagene 10 is significantly enriched in the insulin signaling pathway, response to endogenous stimulatory phosphatase binding, and protein complex.

### 3.7 The human PPI network

The human protein-protein interaction (PPI) network was constructed to detect the most important biological molecules and corresponding protein components, which may play critical tasks in the progress of the ageing brain together with Type 2 Diabetes, by the forecast interactions of identified DEGs. In total 1339 nodes and 11,503 edges were screened through the PPI network (Figure 2A). Ten hub nodes with the highest degrees of DEGs are RHOA, RPLP0, RPL10A, RPS19, LYN, SHC1, PLCB1, PRKCA, EIF4A1, and CDKN1A, shown in Table 4. These DEGs are presented in a classification conferring the number of adjacent nodes and switches in protein encoding genes in the PPI network. The Metagene 9 - PPI network (Figure 2B and Table 5) had protein coding-gene EGR1 (Early Growth Response 1) which is also related to the AGE-RAGE signaling pathway in diabetic complications and Angiopoietin Like Protein 8 Regulatory (ALP8R) Pathway which has closely related with insulin signaling pathway. Functional annotations can be linked to the EGR1 gene including DNA-binding transcription factor activity. The Metagene 2 - PPI network (Figure 2C and Table 5) VIM, AHNAK, YBX3, UBC, S100B, MAGOH, RALA, GFAP, S100A10, and EZR genes which are significantly enriched in astrocyte differentiation (GO:0048708) in BP, focal adhesion (GO:0005925) in CC, and identical protein binding GO:0042802 in MF.

**Table 4:** The top ten genes of PPI network of the temporal cortex of T2D DEGs.

Genes	ND	BC	Expression	log (FC)
RHOA	212	244353.24	6.58071279	0.674
RPLP0	129	69604.44	8.67200301	0.802
RPL10A	117	55849.12	8.45063127	0.669
RPS19	99	48260.67	7.91011409	-0.62
LYN	91	95095.76	4.83679024	-0.661
SHC1	85	63460.66	6.54869794	0.703
PLCB1	82	113344.61	7.98817655	-0.704
PRKCA	75	113728.05	6.70938912	-0.637
EIF4A1	73	52607	5.36867282	0.775
CDKN1A	65	140588.28	6.60256706	0.632
Node Degree, ND; Betweenness Centrality, BC

**Table 5:** The Metagenes 2 and 9 top hub genes of PIP network of the temporal cortex of T2D gene expression set.

Metagenes	Genes	ND	BC	Expression	log (FC)
Metagene 2	VIM	10	111	7.55238552	1.14
	AHNAK	4	61	8.06968982	0.691
	YBX3	4	36	4.9884857	-0.844
	GFAP	1	0	8.46228947	0.935
Metagene 9	EGR1	37	666	7.73370161	0.633

Node Degree, ND; Betweenness Centrality, BC

### 3.8 Validation of the most significant hub genes

**Figure 4:**
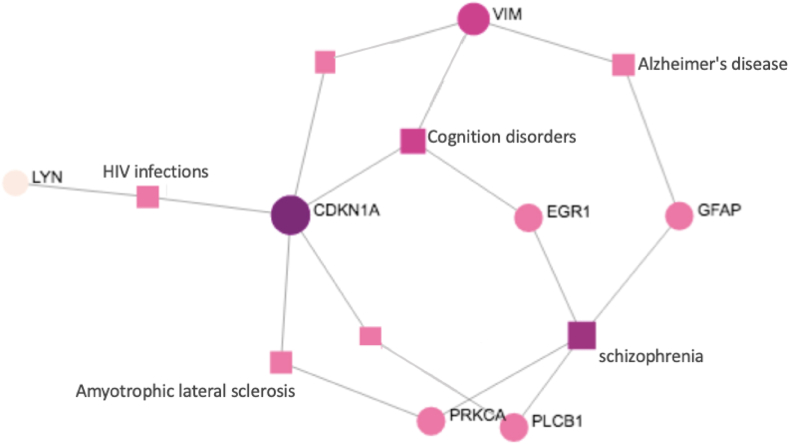
A gene-disease association network of T2D dataset and four neurological disorders of the most significant hub genes at significance level p−value ≤ 0.05.

There were 201 DEGs in total 123 down-regulated and 78 up-regulated at the significance p−value<0.05 and |log(FC)|>0.5 in the GSE54765 dataset. The six hub genes CDKN1A, COL22A1, EIF4A, GFAP, SLC1A1, and VIM were reported as common DEGs in GSE161355 and GSE54765. The results of the examination performed to confirm this importance are shown in Table 6; it was noticed that all of these hub genes were significantly upregulated in both datasets. The study further built a gene-disease association network as illustrated in Figure 4. Metagenes 2 and 9 DEGs combined with all the significant DEGs of the T2D dataset were observed in the intersection of neurological disorder diseases are reported as cognition disorder, schizophrenia, ALS, and AD.

**Table 6:** Base-2 logarithmic values of DEGs of the shared hub genes in T2D and AD.

Datasets	Samples	Genes	Expression	log (FC)	*p* Value
**GSE161355**		CDKN1A	6.602	0.632	0.0041
	6 T2D	COL22A1	4.694	0.85	0.0057
	(AST, BV, NEU)	EIF4A	5.368	0.775	0.0042
	5 Control	GFAP	8.462	0.935	0.0371
		SLC1A1	5.444	0.855	0.0083
		VIM	7.552	1.14	0.0078
**GSE54765**		CDKN1A	4.782	1.079	0.0058
	2 AD	COL22A1	5.636	0.939	0.0229
	(1.25D3)∗	EIF4A	4.567	0.909	0.0207
	2 Control	GFAP	5.542	0.694	0.0323
		SLC1A1	6.784	0.675	0.0342
		VIM	5.894	0.817	0.0201

∗ 1,25 Dihydroxyvitamin D3.

### 3.9 The common hub genes – transcription factor interaction network analysis

The study analyzed the shared hub genes - transcription factor (TF) interactions and identified ten principal monitoring transcription factors, involving CDKN1A, SHC1, COL22A1, SLC1A1, FOXC1, VIM, PRKCA, GFAP, RHOA, and SRF from the network resulted in parameters of the degree and betweenness centrality (BC) (Table 7). The top ten transcription factors constructed on degree values were presented.

**Table 7:** Summary of the transcription factor interaction network enrichment of the shared hub genes of the T2D and AD.

Gene Id	Gene	Degree	BC	Traits	References
1026	CDKN1A	14	380.7	T2D and AD	[61]
6464	SHC1	14	331.14	T2D and AD	[62]
169044	COL22A1	11	240.07	T2D, AD, and HFD∗	[63]
6505	SLC1A1	10	305.03	T2D and AD	[64]
2296	FOXC1	9	398.68	T2D and AD	[8]
7431	VIM	9	264.94	T2D and AD	[65]
5578	PRKCA	8	130.19	T2D and AD	[66]
2670	GFAP	7	187.42	T2D and AD	[65]
387	RHOA	7	109.22	T2D and AD	[67]
6722	SRF	6	159.6	T2D and AD	[66]

∗ High fat-diet.

## 4 Discussion

This study offered a practical and systematic method for detecting links between T2D and AD at the genomic level in the ageing brain samples. Senescence is the foremost threat reason for dementia-related neuron malfunction, alterations in the mechanism, and the emergence of cellular ageing. Several research have demonstrated that people with type 2 diabetes (T2D) have a higher risk of developing AD [1, 2, 5]. Therefore, it is significant to detect subgroups of cases with T2D that may be more likely to be associated with AD.

The established NMF is not appropriate for this assignment in the first place, this study developed a method of the decision of the optimum rank factorization founded on the UIK definition. We have evaluated the NMF algorithm utilizing UIK method as a tool to reveal biologically relevant features in complex gene expression data to observe the genetic and pathologic relation between T2D and AD.

In this investigation, DEGs are extracted in the first step and other steps are performed, including finding metagenes as a result of NMF and analysis of GO enrichment, probing of the PPI network, and KEGG pathway enrichment in the list of metagenes of the DEGs.

The genes that are differentially expressed, do not necessarily form interacting modules, and “placing” edges between them only because they are connected in the STRING database is not a valid task. DEGs are not very useful in the first place because dimension reduction still needs to be performed [68]. There are three main tasks of the analysis conducted here in this study. The first one is screening genes of interest based on their expression movement on the samples [69], the second, selecting the optimal number of ranks (metagenes) essential to the DEGs of the dataset using NMF based on the UIK method [47, 50, 70]. Third, is the construction of a human PPI network which is based on the results of NMF. Finally, to present a ranking for the genes and proteins within a specific metagene, this study discovered the functionality of the patterns by utilizing DAVID and KEGG pathway enrichments.

One of the shortcomings of this project is that genes that have correlated expression patterns with other genes by the control group could likely be strained out. However, the NMF method can detect genes using extracting the relevant genes for each metagene, based on a gene scoring schema implemented by Kim and Park, 2007 [53]; a filtering scheme is selected regarding expression action across the samples. Another limitation of our study is that the “uikNMF” technique is a relatively new method for gene expression analysis and no consensus process has yet emerged. This study designed in-silico so it is lack of experimental validation to confirm our results. The essential pathways enriched in the metagenes were shared hub genes of the T2D and AD. These results would significantly offer to uncover the relationship between these diseases although using different datasets with the relevant disease status of clinical samples would provide better insights.

Although utilizing a multivariate data analysis methodology for gene choice, notable overlaps between genes with Bury et al., 2021 study and this one was recognized [30]. Cellular senescence, insulin signaling pathways, and protein processing in the endoplasmic reticulum were as well involved in the most enriched KEGG pathways in which their research utilized the WGCNA method.

As reported in Table 2, the Jak-STAT signaling pathway is the most significant (with a p-value <0.05, FDR = 1.98E−24) for Metagene 2. The function of JAK-STAT signaling within the central nervous system is reported previously [71]. It is also nominated as a therapeutic target for AD in a recent clinical and empirical study [72]. Consequently, the KEGG enrichment study reported protein processing in the endoplasmic reticulum pathway for Metagene 3. In a previous study, the task of endoplasmic reticulum in amyloid precursor protein processing and operating is discussed in the framework of AD [73]. In other contribution, amassing indication underlines endoplasmic reticulum as a key organelle in AD despite the role of mitochondria which plays as the critical factor in the apoptotic process [74]. Furthermore, cellular senescence pathway is revealed as the most significant pathway in Metagene 5. In a recent review in Nature, it is examined cellular ageing in the framework of AD, and raised the question of which of the processes cellular ageing or AD could happen in the primary place [75].

For Metagene 10, the KEGG pathway enrichments study of T2D gene expression data revealed insulin signaling pathway (pvalue <0.05, FDR = 4.80E−12) as one of the significant pathways among glioma and ErbB signaling pathway. Sato et al., 2011 reported a possible relation between AD and diabetes in a mouse model. Moreover, the study is further reported the insulin signaling is further tangled in the process of ageing and declining with an increase in age [76].

Table 3 shows Metagene 9 is involved in hsa04974:protein digestion and absorption as the most significant (p-value <0.05) KEGG pathway. In a recent study, protein digestion and absorption pathway was reported as one of the most significant pathway was in diabetic complications in the adult neocortex [77]. Table 3 further lists the significant enrichments of Metagene 9 reported GO terms in biological processes (BP) is in brain development (GO:0007420) [78,79]. The most enriched pathway in cellular component (CC) contains apical part of cell (GO:0045177) pathway. A study that employed mRNAs in the brain of human alcoholics found apical part of the cell pathway (down-regulated DEGs) was characteristically linked to neuronal development and physical plasticity (Lewohl et al., 2011). The most enriched pathway in molecular function (MF) uncovered is ion binding (GO:0043167) for Metagene 9. An integrative bioinformatics study of microarray datasets of AD also reported ion binding pathway using a cross-talk analysis [80].

Metagenes 3, 5, and 10 are primarily involved in the Jak-STAT cascade, epidermal growth factor receptor signaling pathway, response to endogenous stimulus, and biological processes respectively. The most significantly enriched gene ontology terms for Metagene 9 are presented in Table 3. Metagene 2 is involved in focal adhesion kinase activity (GO:0004715), SH2 domain binding, and protein tyrosine kinase activity which regulates cellular proliferation, survival, adhesion, and differentiation, and their role is thus strictly regulated. Metagene 9 is involved in ion transport and ion binding, ion metal-binding pathways whereas Metagene 5 is primarily involved in protein kinase binding and cytosol. Although all the other metagenes are stifled, another characteristic of Tables 2 and 3 together is that Metagenes 2, 3, 5, 9, and 10 come out very active in the KEGG pathways linked to the Jak-STAT signaling pathway, protein processing in endoplasmic reticulum cellular senescence, carbohydrate digestion, and absorption, and insulin signaling pathway respectively.

Our study reported 10 hub gene candidates of RHOA, RPLP0, RPL10A, RPS19, LYN, SHC1, PLCB1, PRKCA, EIF4A1, and CDKN1A. RHOA, RPLP0, PRKCA, and EIF4A1 were further reported as the candidate hub genes in a recent study that described the link between T2D and AD separate gene expression datasets [10].

To confirm our results and on what level of T2D is linked with AD, we further observed Alzheimer’s disease gene expression data of GSE54765 genes were significantly up-regulated in both of the datasets. Therefore, aiming these six hub genes; CDKN1A, COL22A1, EIF4A, GFAP, SLC1A1, and VIM would propose insightful advances. We built a gene-disease connection network as demonstrated in Figure 4 to approve the link T2D that leads to AD. The investigation explored additional shared hub genes - transcription factor (TF) interactions and detected ten principal monitoring transcription factors, involving CDKN1A, SHC1, COL22A1, SLC1A1, FOXC1, VIM, PRKCA, GFAP, RHOA, and SRF from the network-based parameters of the degree and betweenness centrality (BC) (Table 7). All of the genes actively involved both in T2D and AD and except COL22A1 are also listed as one of the DEGs in the AD-risk factor gene in the “high fat-diet (HFD)” gene expression dataset GSE68231 which was reported in a previous finding [63].

## 5 Conclusions

NMF analysis of the Type 2 Diabetes of aging brain dataset revealed ten metagenes representing collections of genes with correlated expression patterns concerning the conditions in the dataset. The NMF results with correlation scores and gene expression levels uncovered PPI network interactions within the most highly expressed metagenes. Metagenes 2 and 9 appear to cooperate to suppress other metagenes for the control and diabetic cells while they are active such as metagenes 2, 3, 5, and 10. A metagene PPI network also uncovered similar trends. The NetworkAnalyst tool displayed many impressive specializations of parts among the PPI network of the metagenes, and the analysis presented here would provide valuable insight for proposing associated genes between T2D and AD in prospective studies. The idea of a pathological and genetic link between T2D and AD is supported by our results. In this study, we showed that T2D bonds various shared several progressive biological processes that provide to neuronal dysfunction that might to lead functional impairment. This type of investigation would be helpful for the assembly of genomic evidence-informed suggestions about the precise AD forecast, detection, and improving public awareness of the harmful consequences of T2D on humans.

## Declarations

### Author contribution statement

M.A. Conceptualization, Software, Investigation, Writing – original draft Preparation, Writing – review & editing. K.A. Conceptualization, Methodology, Writing – review & editing. S.A. Conceptualization, Investigation, Writing – review & editing. N.A. Conceptualization, Investigation, Writing – review & editing. I. K. Conceptualization, Data Analysis, Methodology, Software, Investigation, Writing – original draft Preparation, Writing – review & editing. E.G. Conceptualization, Data Analysis, Methodology, Software, Investigation, Writing – original draft Preparation, Writing – review & editing, Visualization.

### Funding statement

Muhammad Afzal was supported by the Deanship of Scientific Research at Jouf University [DSR2022-RG-0150].

### Data availability statement

The GSE161355 and GSE54765 gene expression datasets are utilized in this study are available on the NIH GEO (https://www.ncbi.nlm.nih.gov/geo/query/acc.cgi?acc=GSE161355) and (https://www.ncbi.nlm.nih.gov/geo/query/acc.cgi?acc=GSE54765) public repository.

### Declaration of interest’s statement

The authors declare no conflict of interest.

### Additional information

No additional information is available for this paper.

## Appendix A Supplementary data

Figure S1_V3 Figure S1_V3

Figure S2_V3 Figure S2_V3

Table S1_V3 Table S1_V3

Table S2_V3 Table S2_V3
