# Supplementary material for: Revealing genetic links of Type 2 diabetes that lead to the development of Alzheimer’s disease
Source: Heliyon. 2022 Dec 16;9(1):e12202. doi: 10.1016/j.heliyon.2022.e12202 (PMC9876837; doi:10.1016/j.heliyon.2022.e12202)
Supplement: Figure S1_V3 [file mmc1.pdf]

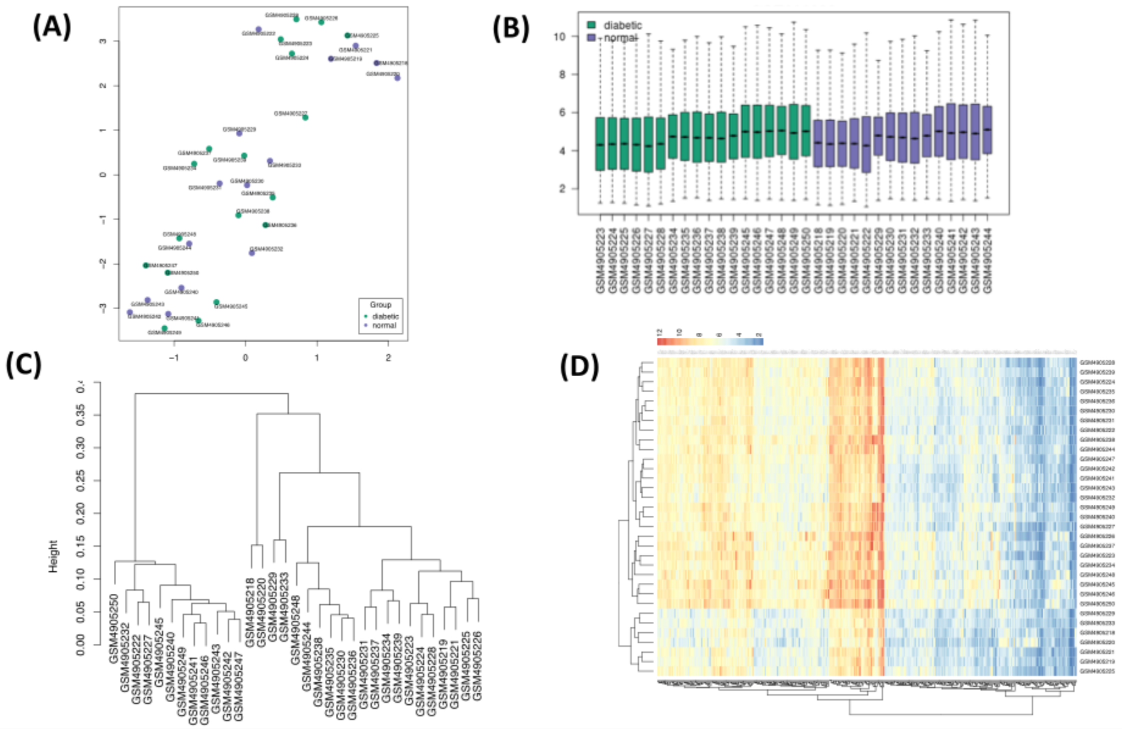

Figure S1. (A) Before processing the GSE161355 microarray gene expression dataset, uniform manifold approximate and projection between control (neurones, astrocytes, and endothelial cells) and Type 2 Diabetic samples is explored to observe the structure in the high-dimensional dataset and for the quality control (QC). **(B)** A boxplot of gene expression samples by expression values of base-2 logarithmic scale is presented. **(C)** A hierarchical clustering map of the dataset. **(D)** The DEGs heatmap of the dataset.
