# Supplementary material for: Revealing genetic links of Type 2 diabetes that lead to the development of Alzheimer’s disease
Source: Heliyon. 2022 Dec 16;9(1):e12202. doi: 10.1016/j.heliyon.2022.e12202 (PMC9876837; doi:10.1016/j.heliyon.2022.e12202)
Supplement: Figure S2_V3 [file mmc2.pdf]

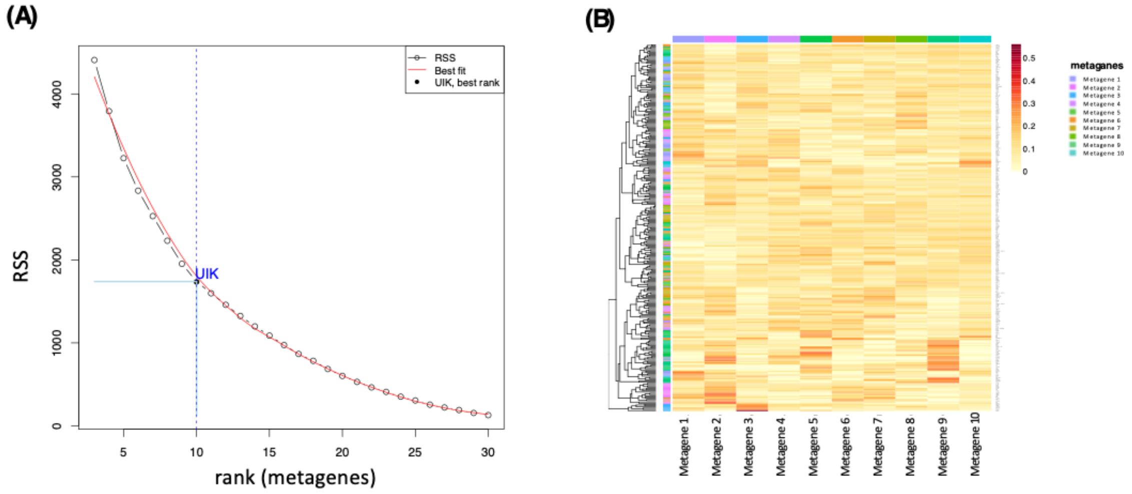

Figure S2. (A) Rank factorization (r) against the Residual Sum of Squares (RSS) curve. The rank factorization values are ranged from 3 to 30 by 1. The optimal (best) rank was detected as $r = 10$ using the UIK method. **(B)** The heatmap of the Metagenes by the official gene symbols.
